# Admissions to intensive care unit of HIV-infected patients in the era of highly active antiretroviral therapy: etiology and prognostic factors

**DOI:** 10.1186/cc10419

**Published:** 2011-08-26

**Authors:** Hou-Hsien Chiang, Chien-Ching Hung, Chang-Min Lee, Hsuan-Yu Chen, Mao-Yuan Chen, Wang-Huei Sheng, Szu-Min Hsieh, Hsin-Yun Sun, Chao-Chi Ho, Chong-Jen Yu

**Affiliations:** 1 Department of Internal Medicine, Far East Memorial Hospital, Nanya South Road, New Taipei City 220, Taiwan; 2 Division of Infectious Diseases, Department of Internal Medicine, National Taiwan University Hospital, Chung-Shan South Road., Taipei 100, Taiwan; 3 Division of Thoracic Surgery, Department of Surgery, National Taiwan University Hospital, Chung-Shan South Road., Taipei 100, Taiwan; 4 Institute of Statistical Science, Academia Sinica, Academia Road Section 2, Taipei 115, Taiwan; 5 Division of Chest Medicine, Department of Internal Medicine, National Taiwan University Hospital, Chung-Shan South Road, Taipei 100, Taiwan

## Abstract

**Introduction:**

Although access to highly active antiretroviral therapy (HAART) has prolonged survival and improved life quality, HIV-infected patients with severe immunosuppression or comorbidities may develop complications that require critical care support in intensive care units (ICU). This study aimed to describe the etiology and analyze the prognostic factors of HIV-infected Taiwanese patients in the HAART era.

**Methods:**

Medical records of all HIV-infected adults who were admitted to ICU at a university hospital in Taiwan from 2001 to 2010 were reviewed to record information on patient demographics, receipt of HAART, and reason for ICU admission. Factors associated with hospital mortality were analyzed.

**Results:**

During the 10-year study period, there were 145 ICU admissions for 135 patients, with respiratory failure being the most common cause (44.4%), followed by sepsis (33.3%) and neurological disease (11.9%). Receipt of HAART was not associated with survival. However, CD4 count was independently predictive of hospital mortality (adjusted odds ratio [AOR], per-10 cells/mm^3 ^decrease, 1.036; 95% confidence interval [CI], 1.003 to 1.069). Admission diagnosis of sepsis was independently associated with hospital mortality (AOR, 2.91; 95% CI, 1.11 to 7.62). A hospital-to-ICU interval of more than 24 hours and serum albumin level (per 1-g/dl decrease) were associated with increased hospital mortality, but did not reach statistical significance in multivariable analysis.

**Conclusions:**

Respiratory failure was the leading cause of ICU admissions among HIV-infected patients in Taiwan. Outcome during the ICU stay was associated with CD4 count and the diagnosis of sepsis, but was not associated with HAART in this study.

## Introduction

After the introduction of highly active antiretroviral therapy (HAART), the life expectancy of HIV-infected patients has significantly increased and the incidence of illnesses associated with AIDS markedly decreased [1]. Nevertheless, HIV-related complications that may require critical care support continue to occur in HIV-infected patients who are unaware of their HIV serostatus and do not initiate HAART and appropriate antimicrobial prophylaxis, or who fail to respond to HAART with virological and immunologic failures. These patients may also require critical care because of other co-morbidities such as hepatitis co-infections, alcoholism, or chronic obstructive pulmonary disease [2]. Although respiratory failure and *Pneumocystis jirovecii *pneumonia have declined in the HAART era compared with the pre-HAART era, they remain the most common diagnoses of HIV-infected patients who were admitted to ICUs [3,4]. Compared with patients in the pre-HAART era, patients in the HAART era are more likely to have life-threatening sepsis, neurologic disorders, and complications of end-stage liver disease [4-6].

Several studies have shown that the advent of HAART not only improved the survival of HIV-infected patients admitted to ICU [7-10], but also changed the etiology of admissions to the ICU whereby fewer patients were admitted to the ICU due to opportunistic infections [10-13]. However, the patient populations included in the studies examining the benefits of HAART are heterogeneous in exposure to HAART, durations of HAART, and timing of HAART [14-20]. The results on survival benefits of HAART are inconsistent across the reported studies in the HIV-infected patients who are already admitted to ICU.

In the era of HAART, prognostic factors of mortality for HIV-infected patients admitted to ICU do not appear to have significant changes [8,12,14-20]. These factors include the severity of acute illness (as assessed by Acute Physiology and Chronic Health Evaluation II (APACHE II) score, Simplified Acute Physiology Score II (SAPS II), or Sequential Organ Failure Assessment (SOFA) score), presence of organ failure (requirement of mechanical ventilator support, shock, renal failure), CD4 lymphocyte count, hospital-to-ICU interval, and serum albumin level. However, these reports mostly came from North America, Latin America, and European countries that enrolled largely white, black, and Hispanic people. It remains unknown whether HIV-infected patients in the Asia-Pacific countries who are admitted to the ICU share the same etiologies and prognostic factors.

In this study, we aimed to describe the etiologies of ICU admissions of HIV-infected patients in a university hospital in Taiwan and to examine the prognostic factors of hospital mortality in the era of HAART. The results of our study will be compared with those of other published studies in HIV-infected patients admitted to ICUs in the HAART era.

## Materials and methods

### Study population

This retrospective cohort study was conducted in the National Taiwan University Hospital, the largest designated hospital to provide inpatient and outpatient HIV care in Taiwan, to enroll all HIV-infected patients aged 18 years or greater who were admitted to the medical and surgical ICU from 1 January, 2001 to 28 February, 2010. The Research Ethics Committee of the hospital approved the study protocol and waived the need for informed consent.

HAART was introduced into Taiwan in April 1997 and all HIV-infected patients have free-of-charge access to antiretroviral therapy according to local treatment guidelines and inpatient and outpatient care that is related to HIV infection at designated hospitals and clinics around Taiwan. The medical costs, including antiretroviral therapy and laboratory investigations such as determinations of CD4 lymphocyte count and plasma HIV RNA load, are totally reimbursed by the National Health Insurance and a special budget of Taiwan Centers for Disease Control. Regarding the end-of-life decisions, patients and/or their family can choose not to receive mechanical ventilation, inotropic agents, cardio-pulmonary-cerebral resuscitation, or electric shock. However, when patients are in critical illness, we do not withdraw mechanical ventilation or inotropic agents that are already applied on them.

### Data collection and definitions

We used a standardized case collection form to record information on demographics, clinical history, risk factors for HIV infection, HAART, and CD4 lymphocyte count and plasma HIV RNA load that were available within one month of ICU admission. If patients were readmitted to the ICU during the same hospitalization, only the data from the first admission were included for analysis of admission diagnoses and mortality predictors.

Prior HAART use was defined as receipt of at least two classes of antiretroviral drugs at the time of hospital admission [21]. Laboratory data within three days of ICU admission (usually within 24 hours) were recorded. Organ failure status (requirement of mechanical ventilator support, renal replacement, or shock) and APACHE II scores on ICU admission were also recorded [22]. Shock status was considered if use of vasopressors was longer than 24 hours. The primary outcome was hospital mortality.

Diagnosis of *P. jirovecii *pneumonia was based on identification of *Pneumocystis *in the sputum, bronchoalveolar-lavage fluid, or transbronchoscopic or surgical lung biopsy [23,24]. For patients admitted to ICU with interstitial pneumonitis that was diagnosed based on radiographic presentations of ground-glass opacities by high-resolution computed tomography (HRCT), but without isolated *P. jirovecii*, they were also classified as having interstitial pneumonitis of unknown etiology.

### Statistical analysis

Clinical characteristics are reported as medians and interquartile range (IQR) or numbers and percentages. Multivariable logistic regression analysis was used to evaluate patient characteristics associated with hospital mortality. Variables with *P *values less than 0.20 in the univariable analyses were entered into a multivariable logistic regression model with non-stepwise method. Final model fit was assessed using the Hosmer-Lemeshow and specification tests. SPSS version 17.0 (SPSS Inc., Chicago, IL, USA) was performed for statistic analyses. *P *values less than 0.05 were considered statistically significant.

## Results

### Demographic and clinical characteristics

During the 10-year-study period, there were 145 ICU admissions for 135 patients. Ten patients were admitted to ICU twice during the same hospitalization. The majority of the ICU cohort were men (91.1%) and Taiwanese (98.5%) (Table 1). The median age was 39 years. Sexual contact was the main risk factor for HIV transmission. The median interval between first HIV diagnosis to ICU admission was 1.23 months (IQR, 0-36 months), and 60 patients (44.4%) were newly diagnosed as having HIV infection on admission. The median plasma HIV viral load that was available for 121 patients was 217,000 copies/ml (IQR, 34,500-631,500 copies/ml), and the median CD4 lymphocyte count that was available for 129 patients was 30 cells/mm^3 ^(IQR, 13-103 cells/mm^3^). Overall, 82 patients (60.7%) received HAART during the ICU stay: 49 patients (36.3%) receiving prior HAART at hospital admission, and 12 patients (8.9%) initiating HAART during the ICU stay.

**Table 1:** Clinical characteristics of 135 HIV-infected patients who were admitted to the ICUs at the National Taiwan University Hospital from 2001 to 2010

Characteristics	135 patients
**Median age (years)**	39 (31-50)
**Male sex**	123 (91.1)
**Race/ethnicity**	
Taiwanese	133 (98.5)
other Asians	2 (1.5)
**HIV risk factor**	
Homosexual	53 (39.3)
Bisexual	7 (5.2)
Injecting drug use	16 (11.9)
Transfusion-related	3 (2.2)
Heterosexual/other/unknown	56 (41.5)
**HIV-related characteristics**	
Newly diagnosed HIV infection	60 (44.4)
Time since HIV diagnosis (month)	1.23 (0-36)
Time since AIDS diagnosis (month)	0.73 (0-26)
Prior HAART at hospital admission	49 (36.3)
Duration of HAART for patients on prior HAART (month)*^a ^*	13 (6-48)
HAART initiated during ICU stay	12 (8.9)
HAART use in ICU	82 (60.7)
HIV viral load (copies/ml)*^b^*	217,000 (34,500-631,500)
CD4 lymphocyte count (cells/mm^3^)*^c^*	30 (13-103)
**Organ failure status**	
Mechanical ventilator use	106 (78.5)
Shock (use of vasopressors > 24 hours)	49 (36.3)
Renal replacement therapy	11 (8.1)
**Time from hospitalization to ICU admission (days)**	2 (0-9.0)
**Laboratory data**	
Albumin (g/dL)*^d^*	2.83 (2.40-3.40)
LDH (units/L)*^e^*	966.5 (686.5-1446.25)
**APACHE II score**	19 (15-25)
**in-ICU mortality**	50 (37.0)
**in-hospital mortality**	66 (48.9)

Values are given as median (interquartile) or number (%), unless otherwise indicated.

*^a^*Data were available for 49 patients with prior HAART at hospital admission.

*^b^*Data were available for 121 patients.

*^c^*Data were available for 129 patients.

*^d^*Data were available for 127 patients.

*^e^*Data were available for 114 patients.

APACHE II, Acute Physiology and Chronic Health Evaluation II; HAART, highly active antiretroviral therapy; LDH, lactate dehydrogenase.

With respect to the organ failure status, 106 patients (78.5%) needed mechanical ventilator support, 49 patients (36.3%) needed vasopressors for more than 24 hours after admission to ICU, and 11 patients (8.1%) received renal replacement therapy during the ICU stay. The median serum albumin level on ICU admission that was available for 127 patients was 2.83 g/dl (IQR, 2.40-3.40 g/dl). The median APACHE II score on ICU admission was 19 (IQR, 15-25). ICU mortality was 50 of 135 patients (37.0%), and hospital mortality was 66 of 135 patients (48.9%).

### Diagnoses of ICU admissions

Respiratory failure was the cause for ICU admissions in 60 of all 135 patients (44.4%; Table 2). Among the patients with respiratory failure, interstitial pneumonitis with ground-glass opacity was the most common cause of respiratory failure (51 patients, 37.8%). *P. jirovecii *pneumonia was confirmed in 11 patients (8.1%), and pathology-proven cytomegalovirus (CMV) pneumonitis occurred in nine patients (6.7%). Sepsis (including bacterial pneumonia) was diagnosed in 45 patients (33.3%), followed by neurological disease (16 patients, 11.9%) and postoperative care (five patients, 3.7%).

**Table 2:** Diagnoses of 135 HIV-infected patients admitted to ICU

Admission diagnosis	Number (%) of admissions
**Respiratory failure**	60 (44.4)
Interstitial pneumonitis with ground glass opacity	51 (37.8)
Pneumocystosis	11 (8.1)
CMV (pathology-proven)	9 (6.7)
Interstitial pneumonitis of unknown etiology	35 (25.9)
Others	11 (8.1)
**Sepsis (including bacteria pneumonia)**	45 (33.3)
**Neurological disease**	16 (11.9)
**Postoperative care**	5 (3.7)
**Trauma**	3 (2.2)
**Metabolic disturbance**	2 (1.5)
**Cardiac disease**	2 (1.5)
**Gastrointestinal bleeding**	2 (1.5)
**Drug overdose**	1 (0.7)
**Miscellaneous**	3 (2.2)

CMV, cytomegalovirus.

### Predictors of in-ICU and in-hospital mortality

**Figure 1:**
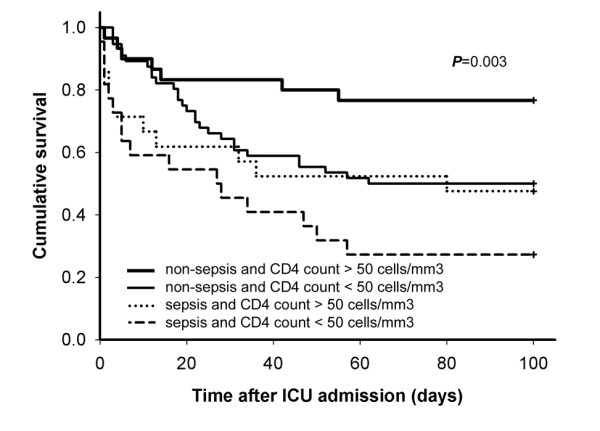
**Kaplan-Meier survival curves stratified by admission diagnosis of sepsis and CD4 lymphocyte count less than 50 cells/mm^3^**. *P *value of log-rank test between the four groups is 0.003.

In the univariable analysis of hospital mortality, there was no difference between survivors and non-survivors with respect to prior HAART, HAART initiated during the ICU stay, or HAART use during the stay in the ICU (Table 3). The factors associated with hospital mortality in univariable analysis were CD4 lymphocyte count (per 10 cells/mm^3 ^decrease), admission diagnosis of sepsis, the interval between hospitalization and ICU transfer of more than 24 hours, and serum albumin level (per 1 g/dl decrease; Table 3). In multivariable logistic regression analysis, the independent predictors of hospital mortality were CD4 lymphocyte count (per 10 cells/mm^3 ^decrease) (adjusted odds ratio (AOR), 1.036; 95% confidence interval (CI), 1.003-1.069; *P *= 0.033), and diagnosis of sepsis (AOR, 2.91; 95% CI, 1.11-7.62; *P *= 0.029). Kaplan-Meier survival curves for 129 patients stratified by sepsis and CD4 count less than 50 cells/mm^3 ^(six patients without available CD4 counts were excluded) are showed in Figure 1. Log-rank testing was significant difference between the four groups (*P *= 0.003).

**Table 3:** Univariable and multivariable analyses of characteristics associated with hospital mortality

Characteristics	Univariable analysis		Multivariable analysis	
	Odds ratio (95% CI)	*P* value	Odds ratio (95% CI)	*P* value
**Age (per 10-year increase)**	1.15 (0.88-1.51)	0.3		
**Male**	2.03 (0.58-7.10)	0.27		
**HIV risk factor**		0.8		
MSM	reference			
Bisexual	0.78 (0.16-3.82)	0.8		
Injecting drug use	0.62 (0.20-1.96)	0.4		
Transfusion-related	2.08 (0.18-24.31)	0.6		
Heterosexual/other/unknown	1.12 (0.53-2.37)	0.8		
**HIV-related characteristics**				
Newly diagnosed HIV infection	1.56 (0.79-3.08)	0.205		
Prior HAART at hospital admission	0.78 (0.39-1.57)	0.5		
HAART initiated during ICU stay	1.05 (0.32-3.44)	0.9		
HAART use in ICU	1.12 (0.56-2.24)	0.7		
HIV viral load (per 1-log copies/ml increase)*^a^*	1.025 (0.83-1.26)	0.8		
CD4 lymphocyte count (per 10-cells/mm^3 ^decrease)*^b^*	1.033 (1.004-1.063)	0.027	1.036 (1.003-1.069)	0.033
**Admission diagnosis**				
Respiratory failure	0.99 (0.50-1.95)	1.0		
Interstitial pneumonitis with ground glass opacity	0.78 (0.39-1.57)	0.5		
Sepsis (including bacterial pneumonia)	2.25 (1.08-4.69)	0.03	2.91 (1.11-7.62)	0.029
**Hospitalization to ICU admission > 24 hours**	2.72 (1.23-6.01)	0.013	2.21 (0.90-5.47)	0.085
**Albumin (per 1 g/dl decrease)***^c^*	1.69 (1.04-2.74)	0.034	1.31 (0.74-2.34)	0.36

The goodness of fit (Hossmer-Lemeshow Chisquare *P *value) was 0.619. 14 patients were not included in the multivariable model because of missing data.

*^a^*Data were available for 121 ICU admissions.

*^b^*Data were available for 129 ICU admissions.

*^c^*Data were available for 127 ICU admissions.

CI, confidence interval; HAART, highly active antiretroviral therapy; MSM, men who have sex with men.

## Discussion

This study is the first cohort study to analyze the etiologies of ICU admissions and to investigate the predictors of mortality in HIV-infected patients in the Asia-Pacific region. In the current study, respiratory failure was the leading cause of ICU admissions among HIV-infected patients, followed by sepsis and neurological disease. CD4 lymphocyte count and admission diagnosis of sepsis are two independent predictors of hospital mortality in multivariable analysis.

The causes of ICU admissions observed in our study are not different from those observed in other studies that were conducted in North America, Latin America, and European countries [8,10,14-16,18-20,25] (Table 4). However, the percentage of each etiology differs between our study and others. In our study, 8.1% of the patients admitted to ICU had diagnoses of *P. jirovecii *pneumonia, and 25.9% of the patients were classified as interstitial pneumonitis with unknown etiology, which might be probable cases of *P. jirovecii *pneumonia when the radiographic findings and clinical response to specific anti-pneumocystosis therapy were taken into consideration. Taking definite and probable cases of pneumocystosis together, it might comprise 34.0% of the patients, which is more than those of other reports [8,10,14-16,18-20,25] (Table 4). In addition, the percentage of newly diagnosed HIV infection on admission (46.2%) is higher, and the median CD4 count (30 cells/mm^3^) is lower than those in other studies (Table 4). The above observations may be because our hospital is a referral hospital and the majority of patients admitted to this hospital were severely immunocompromised and were not on HAART [26,27]. In our study, 63.7% of the patients did not receive HAART before hospital admissions.

**Table 4:** Comparisons of studies regarding HIV-infected patients admitted to ICU in the HAART era

City [reference]	San Francisco [8]	**San Francisco **[16]	New York [20]	Paris [18]	Mexico [14]	São Paulo [15]	Rio de Janeiro [19]	Taipei
**Study year**	1996-1999	2000-2004	1997-1999	1996-2005	1996-2006	1996-2006	2006-2008	2001-2010
**HIV-related characteristics**								
Newly diagnosed HIV (%)	5.6	-	-	19.7^*a*^	26	38	28	44.4
Median CD4 count (cells/mm^3^)	64	109	85	-	-	39	75	30
**ICU admission diagnosis (%)**								
Respiratory failure (%)	40.7	42.3	30.0	58.8	51.0	33.1	29	44.4
pneumocystosis (%)	10.7	13.8	-	18.7	-	23.2	-	8.1^*b*^
Sepsis (%)	11.9	20.3	13.0	23.9	26.0	31.2	20.5	33.3
Neurological disease (%)	12.4	16.3	18.0	32	15.0	19.4	22.7	11.9
Others (%)	35.0	21.1	39.0	-	21.0	16.2	27.3	13.3
**Mortality predictors**								
ART use	No prior HAART univariably increased hospital mortality, 1.8 (1.02-3.2), but not significantly in multivariable analysis	No association	No association	No association	No prior HAART independently increased ICU mortality, 3.33 (1.43-10.0)*^c^*	No ART use in ICU independently increased 6-month mortality, 2.00 (1.41-2.86)	No association	No association
CD4 count (cells/mm3)	-	-	CD4 < 200 univariably increased hospital mortality, 2.24 (1.16-4.31), but not significantly in multivariable analysis	No association	-	CD4 < 50 independently increased ICU mortality, 2.10 (1.17-3.76)	No association	CD4 (per 10-cells/mm^3 ^decrease) independently increased hospital mortality, 1.036 (1.003-1.069)
Admission diagnosis of sepsis	-	No significant difference between with sepsis and respiratory failure	-	Severe sepsis independently increased ICU mortality, 3.67 (1.53-8.80)	Septic shock independently increased ICU mortality, 2.4 (1.1-5.2)*^c^*	Sepsis independently increased ICU mortality, 3.16 (1.65-6.06)	Severe sepsis/septic shock independently increased 28-day mortality, 3.13 (1.21-8.07)*^c ^*	Sepsis independently increased hospital mortality, 2.91 (1.11-7.62)
Hospital-to-ICU interval	-	-	-	Delayed ICU admission independently increased ICU mortality, 3.04 (1.29-7.71)	-	-	-	Hospital-to-ICU interval > 24 hours univariably increased hospital mortality, 2.72 (1.23-6.01), but not significantly in multivariable analysis
Serum albumin level (g/dL)	Serum albumin < 2.6 independently increased hospital mortality, 3.5 (1.8-6.6)	Lower serum albumin (per 1-g/dl decrease) independently increased hospital mortality, 2.08 (1.41-3.06)	-	-	No association	No association	-	Lower serum albumin (per 1-g/dl decrease) univariably increased hospital mortality, 1.69 (1.04-2.74), but not significantly in multivariable analysis

Values are given as odds ratio (95% confidence interval), unless otherwise indicated.

*^a^*HIV diagnosis within 60 days before ICU admission.

*^b^*Diagnosis was based on identification of *Pneumocystis *in the sputum, bronchoalveolar-lavage fluid, or transbronchoscopic or surgical lung biopsy.

*^c^*Values are given as hazard ratio (95% confidence interval).

ART, antiretroviral therapy; HAART, highly active antiretroviral therapy.

In this study, we identified two independent predictors of hospital mortality in multivariable analysis: CD4 lymphocyte count and admission diagnosis of sepsis. Low CD4 lymphocyte count is associated with suppressed cellular immunity, and its association with increased mortality has also been demonstrated in prior studies [15,20].

The association between sepsis and higher rate of mortality in the critically ill HIV-AIDS population has been shown in previous studies [14,15,18,19] (Table 4). Japiassú AM et al. reported that sepsis is a major determinant of 28-day and six-month mortality in HIV-infected patients admitted to ICU (adjusted hazard ratio, 3.13 and 3.35, respectively). The above study and our study both revealed that pneumonia is the most common site of infection (52% and 55.6%, respectively), whereas our cohort had cases of fewer primary bacteremia (15.6%) and nosocomial infections (37%) [19]. The screening of HIV infection and high suspicion to sepsis diagnosis could contribute to decrease mortality in critically ill HIV/AIDS patients [19,28,29]. The trend of relatively lower mortality for patients with interstitial pneumonitis with ground-glass opacity may be attributed to increased alertness to identification of severely immunocompromised patients who present with characteristic radiographic findings [1,4].

Although early initiation of HAART has been shown to improve survival in patients with AIDS-related opportunistic infections [13], it remains debatable whether initiation of HAART improves the outcome of patients admitted to ICU owing to factors related to issues of toxicity, bioavailability, and drug-drug interactions [1]. Several studies, including ours, failed to demonstrate short-term survival benefit of HAART in those patients admitted to ICU [16-20], probably because in those patients with severely depleted CD4 counts, improvement of immunity in terms of increase of CD4 counts or function cannot be achieved after short term of HAART. Furthermore, early initiation of HAART in patients with depleted CD4 counts and opportunistic illnesses, such as tuberculosis, non-tuberculous mycobacteriosis, cryptococcosis, and Kaposi's sarcoma, is associated with increased risk of immune reconstitution inflammatory syndrome [13,30], which may increase difficulties in clinical management and mortality in resource-limited settings.

Prevalence and trends of antiretroviral drug resistance mutations may also affect the treatment response to HAART [31]. In Taiwan, the frequency of HIV-1 isolates harbored one or more primary mutations associated with antiretroviral resistance to reverse-transcriptase inhibitors or protease inhibitors increased significantly from 6.6% in 1999 to 2003 to 12.7% in 2004 to 2006 (*P *= 0.003) [32]. In our study, 49 patients had been taking HAART for a median duration of 13 months (IQR, 6-48 months), and their median CD4 count was 67 cells/mm^3 ^(IQR, 16-207 cells/mm^3^). The suboptimal treatment responses to HAART in these patients may be due to the presence of transmitted drug resistance of the HIV strains, poor adherence to antiretroviral therapy prescribed that may result in emergence of antiretroviral drug resistance mutations, and immunologic non-response [31].

Lower serum albumin level has been found to be a poor prognostic factor in the studies from San Francisco General Hospital [8,16]. In our study, we also found that serum albumin level less than 2.8 g/dl is associated with hospital mortality, although it is of no statistical significance in multivariable analysis. Low serum albumin level may be the results of poor nutritional status and severe catabolism from the prolonged critical illness, which may lead to insufficient intravascular volume, hemodynamic instability, increased extravascular lung water, and therefore, increased mortality [33,34].

There are several limitations in this study. First, our study is retrospective and observational in study design, which may preclude us from identifying every possible confounding factor. Second, the case number in this study remains small and prospective studies of larger case number are needed to examine the impact of HAART and other parameters on the outcomes of HIV-infected patients who require care in the ICU. Third, the majority of our patients acquired HIV through sexual contact and our findings may not be generalizable to the health care facilities that provide HIV care to a higher proportion of injecting drug users who are more likely to develop bacterial complications due to use of contaminated syringes, needles and diluent. Fourth, our findings may not be generalizable to other hospitals because the decisions of admissions to ICU in this referral hospital for HIV care depends on clinical assessment of the treating physicians and consultations with the critical care specialists, which may vary among the hospitals providing HIV care. Finally, we were not able to assess the adherence to antiretroviral therapy prescribed in those patients who had been on HAART prior to hospital admission, nor were we able to assess the bioavailability of HAART that was initiated and continued in those patients during the ICU stay.

## Conclusions

In conclusion, we found respiratory failure remained the most common cause of ICU admission for HIV-infected patients in the era of HAART. The hospital mortality of HIV-infected patients who were admitted to the ICU was associated with low CD4 lymphocyte count and the diagnosis of sepsis.

## Key messages

• Respiratory failure is the most common cause of ICU admission for HIV-infected patients in the era of HAART, followed by sepsis and neurological disease.

• The hospital mortality of HIV-infected patients who are admitted to the ICU is associated with low CD4 lymphocyte count and the diagnosis of sepsis.

• Low serum albumin level and delayed ICU admission are associated with poor outcome.

## Abbreviations

AOR: adjusted odds ratio; APACHE II: Acute Physiology and Chronic Health Evaluation II; CI: confidence interval; CMV: cytomegalovirus; HAART: highly active antiretroviral therapy; HRCT: high-resolution computed tomography; IQR: interquartile range; SAPS II: Simplified Acute Physiology Score II; SOFA: Sequential Organ Failure Assessment.

## Competing interests

The authors declare that they have no competing interests.

## Authors' contributions

HHC participated in the design of the study, collected data, performed the statistical analysis, and drafted the manuscript. CCH participated in the design of the study, analyzed and interpreted data, and revised the manuscript. CHL acquired surgical samples, and helped to collect data. HYC performed the statistical analysis, and helped to revise the manuscript. MYC, WHS, SMH, and HYS participated in clinical evaluation of patients, and helped to collect data. CCH conceived of the study, participated in its design and coordination, and revised the manuscript. CJY participated in the design and coordination of the study. All authors read and approved the final manuscript.
